# Supplementary material for: The selfish yeast plasmid exploits a SWI/SNF-type chromatin remodeling complex for hitchhiking on chromosomes and ensuring high-fidelity propagation
Source: PLoS Genet. 2023 Oct 9;19(10):e1010986. doi: 10.1371/journal.pgen.1010986 (PMC10586699; doi:10.1371/journal.pgen.1010986)
Supplement: S1 Table — The genotypes of the yeast strains used in the present study along with the figures depicting the experimental results obtained with them are listed. Strains containing or lacking the native 2-micron plasmid are indicated as [Cir+] or [Cir0], respectively. (DOCX) [file pgen.1010986.s001.docx]

**S1 Table.**

| **Strains** | **Genotype/salient features** | **Relevant**  **Figures** |
| --- | --- | --- |
| CMY685 | *MATa* *ade2-1 his3-11::*P*_GAL10_-REP1-*P*_GAL1_-REP2::HIS3 leu2-3,112 trp1 ura3-1* [Cir^0^] | Fig 1, S1 Fig, Fig 2, S3 Fig |
| CMY687 | *MATa* *ade2-1 his3-11::*P*_GAL10_-3HA-REP1-*P*_GAL1_-REP2-10Myc::HIS3 leu2-3,112 trp1 ura3-1* [Cir^0^] | Fig 1, S1 Fig, Fig 2, S3 Fig |
| CMY689 | *MATa* *ade2-1 his3-11::*P*_GAL1_-REP1-10Myc-*P*_GAL10_-3HA-REP2::HIS3 leu2-3,112 trp1 ura3-1* [Cir^0^] | Fig 1, S1 Fig, Fig 2, S3 Fig |
| CMY699 | *MATa* *ade2-1 his3-11::*P*_GAL1_-FLP(Y343F)-10Myc::HIS3 leu2-3,112 trp1::*4x (half*-FRT*) *ura3-1* [Cir^0^] | Fig 1, S1 Fig, Fig 2, S3 Fig |
| CMY717 | *MATa* *pADE2-REP1-10Myc ade2-1 his3-11 leu2-3,112 trp1 ura3-1* [Cir^0^] | S2 Fig |
| CMY741 | *MATa* *pADE2-REP2-10Myc ade2-1 his3-11 leu2-3,112 trp1 ura3-1* [Cir^0^] | S2 Fig |
| CMY781 | *MATa* *pADE2 ade2-1 his3-11 leu2-3,112 trp1 ura3-1* [Cir^0^] | S4A Fig |
| CMY782 | *MATa* *pADE2 ade2-1 his3-11 leu2-3,112 trp1 ura3-1 rsc2Δ::URA3* [Cir^0^] | S4A Fig |
| CMY783 | *MATa pADE2* *ade2-1 his3-11 leu2-3,112 trp1 ura3-1 truncated rsc2* [Cir^0^] | S4A Fig |
| CMY795 | *MATa* *ade2-1 his3-11::*P*_GAL10_-3HA-REP1-*P*_GAL1_-REP2-10Myc::HIS3 leu2-3,112 trp1 ura3-1 rsc2Δ::URA3* [Cir^0^] | Fig 4,  S4B-S4E Fig,  Fig 5,  Fig 6,  S5 Fig |
| CMY797 | *MATa* *ade2-1 his3-11::*P*_GAL10_-3HA-REP1-*P*_GAL1_-REP2-10Myc::HIS3 leu2-3,112 trp1 ura3-1 truncated rsc2* [Cir^0^] | Fig 4,  S4B-S4E Fig,  Fig 5,  Fig 6,  S5 Fig |
| CMY799 | *MATa* *ade2-1 his3-11::*P*_GAL1_-REP1-10Myc-*P*_GAL10_-3HA-REP2::HIS3 leu2-3,112 trp1 ura3-1 rsc2Δ::URA3* [Cir^0^] | Fig 4,  S4B-S4E Fig,  Fig 5,  Fig 6,  S5 Fig |
| CMY801 | *MATa* *ade2-1 his3-11::*P*_GAL1_-REP1-10Myc-*P*_GAL10_-3HA-REP2::HIS3 leu2-3,112 trp1 ura3-1 truncated rsc2* [Cir^0^] | Fig 4,  S4B-S4E Fig,  Fig 5,  Fig 6,  S5 Fig |
| Y2HGold | *MATa* *trp1-901 leu2-3,112 ura3-52 his3-200 gal4Δ gal80Δ LYS2::GAL1_UAS_–Gal1_TATA_–His3 GAL2_UAS_–Gal2_TATA_–Ade2 URA3::MEL1_UAS_–Mel1_TATA_ AUR1-C MEL1* [Cir^+^] | Fig 7, S6 Fig |
| Y187 | *MATα ura3-52 his3-200 ade2-101 trp1-901 leu2-3,112 gal4Δ gal80Δ met– URA3::GAL1_UAS_–Gal1_TATA_–LacZ, MEL1* [Cir^+^] | Fig 7, S6 Fig |
| CMY891 | *MATa* *trp1-901 leu2-3,112 ura3-52 his3-200 gal4Δ gal80Δ LYS2::GAL1_UAS_–Gal1_TATA_–His3 GAL2_UAS_–Gal2_TATA_–Ade2 URA3::MEL1_UAS_–Mel1_TATA_ AUR1-C MEL1* [Cir^0^] | Fig 7, S6 Fig |
| CMY893 | *MATα ura3-52 his3-200 ade2-101 trp1-901 leu2-3,112 gal4Δ gal80Δ met– URA3::GAL1_UAS_–Gal1_TATA_–LacZ MEL1* [Cir^0^] | Fig 7, S6 Fig |
| CMY861 | *MATα ura3-52::*P*_GAL10_-3HA-REP1-*P*_GAL1_-REP2-10Myc::URA3 trp1 leu2*Δ*1 his3Δ200 pep4::HIS3 prb1Δ1.6R can1 RSC2-SGGGG-CBP-TEV-ProtA-ProtA::TRP1* [Cir^+^] | Fig 9 |
| CMY859 | *MATα ura3-52::*P*_GAL10_-3HA-REP1-*P*_GAL1_-REP2-10Myc::URA3 trp1 leu2Δ1 his3Δ200 pep4::HIS3 prb1Δ1.6R can1 RSC1-SGGGG-CBP-TEV-ProtA-ProtA* [Cir^+^] | S10 Fig |
| NPY7 | *MATα ura3-52::*P*_GAL10_-3HA-REP1-*P*_GAL1_-REP2-10Myc::URA3 trp1 leu2Δ1 his3Δ200 pep4::HIS3 prb1Δ1.6R can1 RSC2-SGGGG-CBP::TRP1* [Cir^+^] | S9 Fig |
| NPY9 | *MATα ura3-52::*P*_GAL10_-3HA-REP1-*P*_GAL1_-REP2-10Myc::URA3 trp1 leu2Δ1 his3Δ200 pep4::HIS3 prb1Δ1.6R can1 RSC1-SGGGG-CBP::TRP1* [Cir^+^] | S11 Fig |
| SGY11081 | *MATa/α ade2-1 trp1-1 can1-100 leu2-3,112 his3-11 ura3-1* [Cir^+^] | Fig 10,  S12-S15 Fig |
| SGY11082 | *MAT*a/α *ade2-1 trp1-1 can1-100 leu2-3,112 ura3-1 RSC2-6HA::URA3/RSC2-6HA::URA3 his3-11::P_GAL1_-REP1-10Myc::HIS3* [Cir^+^] | Fig 10, S12 Fig |
| SGY11083 | *MAT*a/α *ade2-1 trp1-1 can1-100 leu2-3,112 ura3-1 RSC2-6HA::URA3/RSC2-6HA::URA3 his3-11::*P*_GAL1_-REP1-10Myc::HIS3* [Cir^0^] | S14 Fig |
| SGY11084 | *MAT*a/α *ade2-1 trp1-1 can1-100 leu2-3,112 ura3-1 RSC2-6HA::URA3/RSC2-6HA::URA3 his3-11::P_GAL1_-REP2-10Myc::HIS3* [Cir^+^] | Fig 10, S12 Fig |
| SGY11085 | *MAT*a/α *ade2-1 trp1-1 can1-100 leu2-3,112 ura3-1 RSC2-6HA::URA3/RSC2-6HA::URA3 his3-11::*P*_GAL1_-REP2-10Myc::HIS3* [Cir^0^] | S14 Fig |
| SGY11086 | *MAT*a/α *ade2-1 trp1-1 can1-100 leu2-3,112 ura3-1 RSC1-6HA::KanMX4/RSC1-6HA::KanMX4 his3-11::*P*_GAL1_-REP1-10Myc::HIS3* [Cir^+^] | Fig 10, S12 Fig |
| SGY11087 | *MAT*a/α *ade2-1 trp1-1 can1-100 leu2-3,112 ura3-1 RSC1-6HA::KanMX4/RSC1-6HA::KanMX4 his3-11::*P*_GAL1_-REP1-10Myc::HIS3* [Cir^0^] | S14 Fig |
| SGY11088 | *MAT*a/α *ade2-1 trp1-1 can1-100 leu2-3,112 ura3-1 RSC1-6HA::KanMX4/RSC1-6HA::KanMX4 his3-11::*P*_GAL1_-REP2-10Myc::HIS3* [Cir^+^] | Fig 10, S12 Fig |
| SGY11089 | *MAT*a/α *ade2-1 trp1-1 can1-100 leu2-3,112 ura3-1 RSC1-6HA::KanMX4/RSC1-6HA::KanMX4 his3-11::*P*_GAL1_-REP2-10Myc::HIS3* [Cir^0^] | S14 Fig |
| SGY11090 | *MAT*a/α *ade2-1 trp1-1 can1-100 leu2-3,112 ura3-1 his3-11 RSC2-6HA::URA3 RSC1-9Myc::KanMX4* [Cir^+^] | S13 Fig |
| SGY11091 | *MAT*a/α *trp1-1 can1-100 leu2-3,112 his3-11 ura3-1 RSC2-9Myc::KanMX4/RSC2-9Myc::KanMX4 ade2-1::GFP-LacI::ADE2/ade2-1::GFP-LacI::ADE2* [Cir^+^] | Fig 10, S12 Fig |
| SGY11092 | *MAT*a/α *trp1-1 can1-100 leu2-3,112 his3-11 ura3-1 RSC2-9Myc::KanMX4/RSC2-9Myc::KanMX4 ade2-1::GFP-LacI::ADE2/ade2-1::GFP-LacI::ADE2* [Cir^0^] | S14 Fig |
| SGY11093 | *MAT*a*/*α *trp1-1 can1-100 leu2-3,112 his3-11 ura3-1 RSC1-9Myc::KanMX4/RSC1-9Myc::KanMX4 ade2-1::GFP-LacI:: ADE2/ade2-1::GFP-LacI::ADE2* [Cir^+^] | Fig 10, S12 Fig |
| SGY11094 | *MAT*a*/*α *trp1-1 can1-100 leu2-3,112 his3-11 ura3-1 RSC1-9Myc::KanMX4/RSC1-9Myc::KanMX4 ade2-1::GFP-LacI:: ADE2/ade2-1::GFP-LacI::ADE2* [Cir^0^] | S14 Fig |
| SGY11095 | *MAT*a/α *can1-100 leu2-3,112 trp1-1* P*_GAL1_-NDT80::TRP1* P*_GDP1_-GAL4-ER RSC2-6HA::KanMX4 his3-11::* P*_GAL1_-REP1-10Myc::HIS3 ura3-1::*P*_GAL1_-REP2::URA3* [Cir^0^] | S15 Fig |
| SGY11096 | *MAT*a/α *can1-100 leu2-3,112 trp1-1* P*_GAL1_-NDT80::TRP1 P_GPD1_-Gal4-ER RSC1-6HA::KanMX4 his3-11::*P*_GAL1_-REP2-10Myc::HIS3 ura3-1::*P*_GAL1_-REP1::URA3* [Cir^0^] | S15 Fig |
| SGY11099 | *MAT*a *trp1-1 can1-100 his3-11 tRNA-Val::gRNA-TetO leu2-3,112::TetR-tdTomato::LEU2 ade2-1::GFP-LacI-ADE2 ura3::P_GAL10_-REP1-P_GAL1_-REP2::URA3* [Cir^0^] | Fig 11 |
| SGY11100 | *MAT*a *trp1-1 can1-100 his3-11 ura3-1 tRNA-Val::gRNA-TetO leu2-3,112::TetR-tdTomato::LEU2 ade2-1::GFP-LacI-ADE2* [Cir^0^] | Fig 11 |
| SGY11097 | *MAT*a *trp1-1 can1-100 leu2-3,112 his3-11 ura3-1 ade2-1::GFP-LacI::ADE2* [Cir^0^] | Fig 12 |
| SGY11098 | *MAT*a *trp1-1 can1-100 leu2-3,112 his3-11 ura3-1 ade2-1::GFP-LacI::ADE2 SFH1::SFH1-LacI* [Cir^0^] | Fig 12 |
