# Supplementary material for: The selfish yeast plasmid exploits a SWI/SNF-type chromatin remodeling complex for hitchhiking on chromosomes and ensuring high-fidelity propagation
Source: PLoS Genet. 2023 Oct 9;19(10):e1010986. doi: 10.1371/journal.pgen.1010986 (PMC10586699; doi:10.1371/journal.pgen.1010986)
Supplement: S2 Table — The plasmids listed below as groups I-III were used for yeast two-hybrid analyses, expression in E. coli and interaction assays, and cell biological/fluorescence microscopy experiments in yeast, respectively. The figures containing experimental results to which they contributed are indicated. For previously described plasmids, the relevant references are given. (DOCX) [file pgen.1010986.s002.docx]

**S2 Table.**

| **Plasmids** | **Relevant Figures** |
| --- | --- |
| **Group I** | |
| pGBKT7 (DNA binding domain vector in two-hybrid assays) | Fig 7, S6 Fig |
| pGADT7 (activation domain vector in two-hybrid assays) | Fig 7, S6 Fig |
| pGBKT7-*REP1* | Fig 7, S6 Fig |
| pGADT7-AD-*REP1* | Fig 7, S6 Fig |
| pGBKT7-*REP2* | Fig 7, S6 Fig |
| pGADT7-AD-*REP2* | Fig 7, S6 Fig |
| pGBKT7-*SFH1* | Fig 7, S6 Fig |
| pGADT7-*SFH1* | Fig 7, S6 Fig |
| pGBKT7-Sfh1(Δ1-278) | Fig 7, S6 Fig |
| pGADT7-Sfh1(Δ1-278) | Fig 7, S6 Fig |
| **Group II** | |
| pET28a-His6-*SFH1* | Fig 8 |
| pET28a-His6-Sfh1(Δ1-278) | S7 Fig |
| pGEX2T-*REP1* | Fig 8 , S7 Fig |
| pGEX2T-*REP2* | Fig 8 , S7 Fig |
| pGEX2T-GST-*REP2*- reverse P-tac-3HA-*REP1* | S8 Fig |
| **Group III** | |
| pSV1-*ORI*-*STB* [LacO]_256_ (*LEU2*) | Figs S12 and S14 (Mehta *et al*. 2002 J Cell Biol 158: 625-637) |
| pBM272-*REP1* (*ARS*-*CEN* P*_GAL10_*-*REP1*) | S15 Fig (Yang *et al*. 2004 Mol Cell Biol: 24: 5290-5303) |
| pBM272-*REP2* (*ARS*-*CEN* P*_GAL10_*-*REP2*) | S15 Fig (Yang *et al*. 2004 Mol Cell Biol: 24: 5290-5303) |
| pSG1: P*_GAL1_*-*CEN3-STB-ORI* cloned in pSV5) | Fig 11-12 (Ghosh *et al*. 2010 PNAS 104: 13034-13039; Mehta *et al*. 2002 J Cell Biol 158: 625-637) |
| pSV5: *ORI*-*STB* [LacO]_256_ (*TRP1*) | Fig 12 (Mehta *et al*. 2002 J Cell Biol 158: 625-637) |
