# Supplementary figures and images for: The selfish yeast plasmid exploits a SWI/SNF-type chromatin remodeling complex for hitchhiking on chromosomes and ensuring high-fidelity propagation
Source: PLoS Genet. 2023 Oct 9;19(10):e1010986. doi: 10.1371/journal.pgen.1010986 (PMC10586699; doi:10.1371/journal.pgen.1010986)

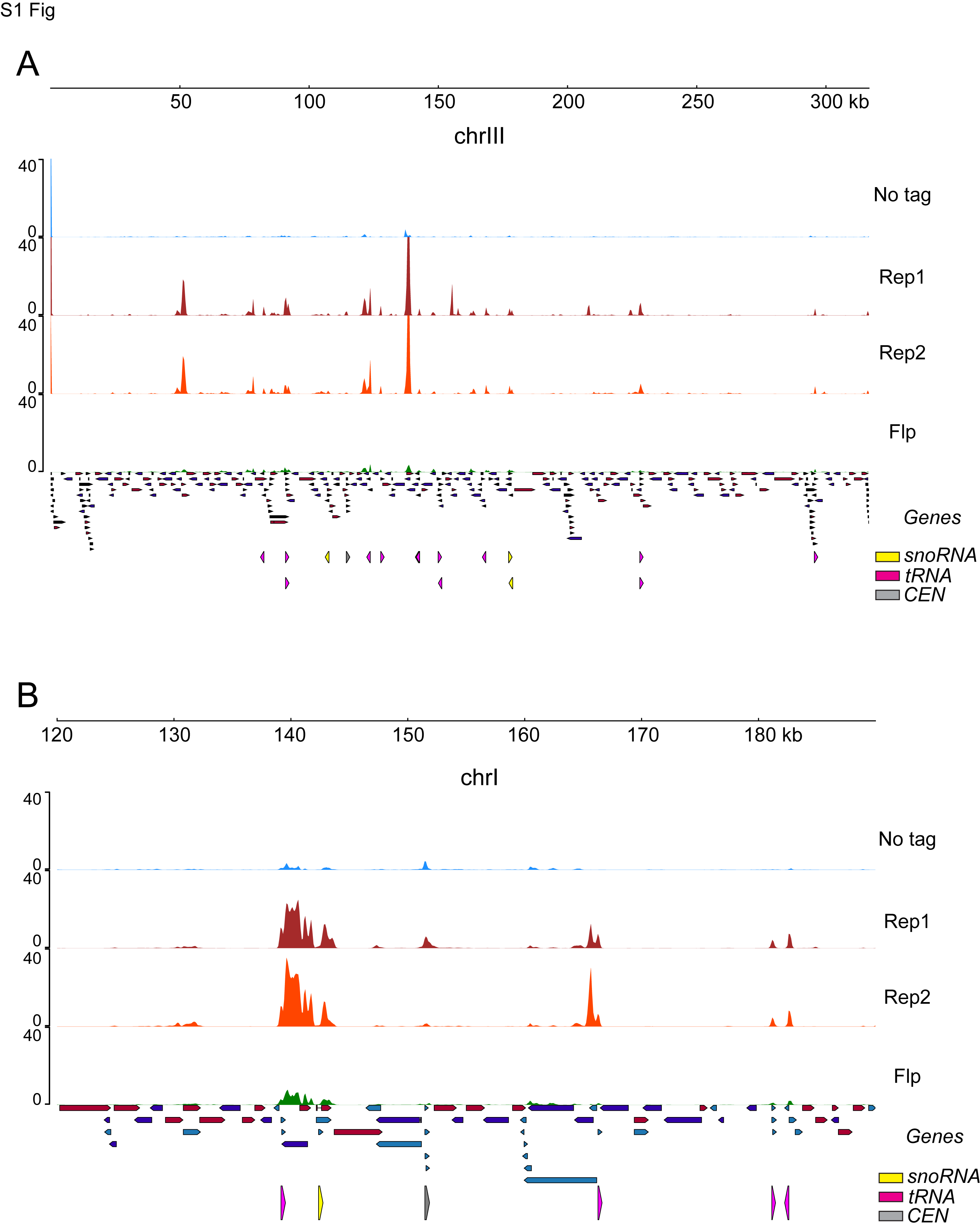

Supplement: S1 Fig — Binding of Rep1 and Rep2 assayed by ChIP-seq is shown using input-corrected signal tracks along with negative controls (No tag and Flp). The locations of snoRNAs, tRNAs and CEN are shown below each track using the colors indicated. (A) chrIII. (B) A region of chrI. (TIF) [file pgen.1010986.s003.tif]

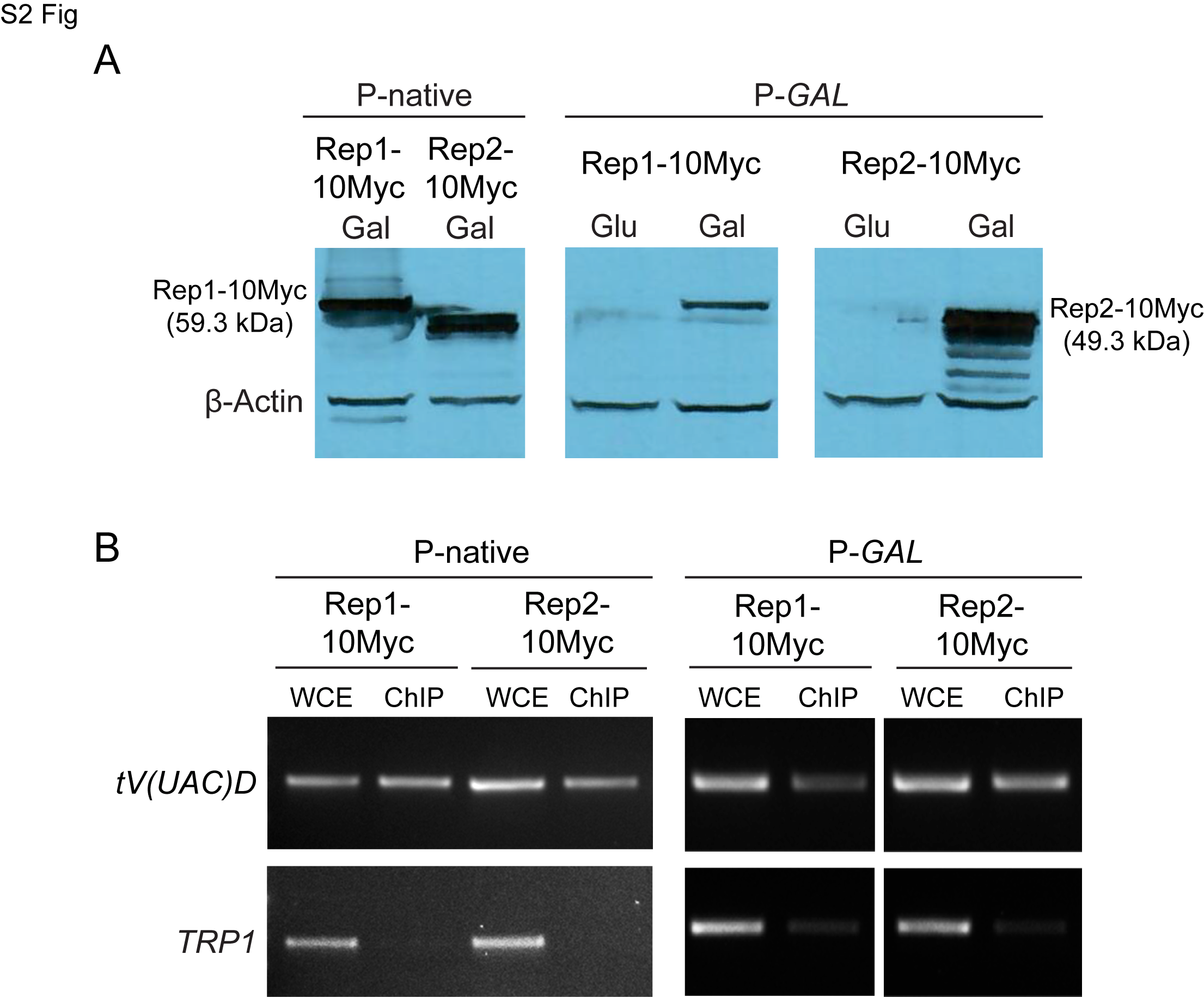

Supplement: S2 Fig — (A) Western blot analysis of strains expressing epitope-tagged Rep1 or Rep2 under the control of their native promoters on the 2-micron plasmid or the GAL1-10 promoter. Gal and Glu refer to growth in medium with galactose or glucose as the carbon source. Blots were probed with anti-Myc (to visualize Rep1/Rep2) and β-actin (loading control) antibodies. (B) ChIP-PCR analysis of Rep1 and Rep2 localization at the tV(UAC)D locus (tRNA valine). WCE refers to the whole-cell extract, or input sample before ChIP, while ChIP refers to the ChIP sample with anti-Myc. PCR was performed for the tRNA valine locus and for a negative control locus (TRP1). (TIF) [file pgen.1010986.s004.tif]

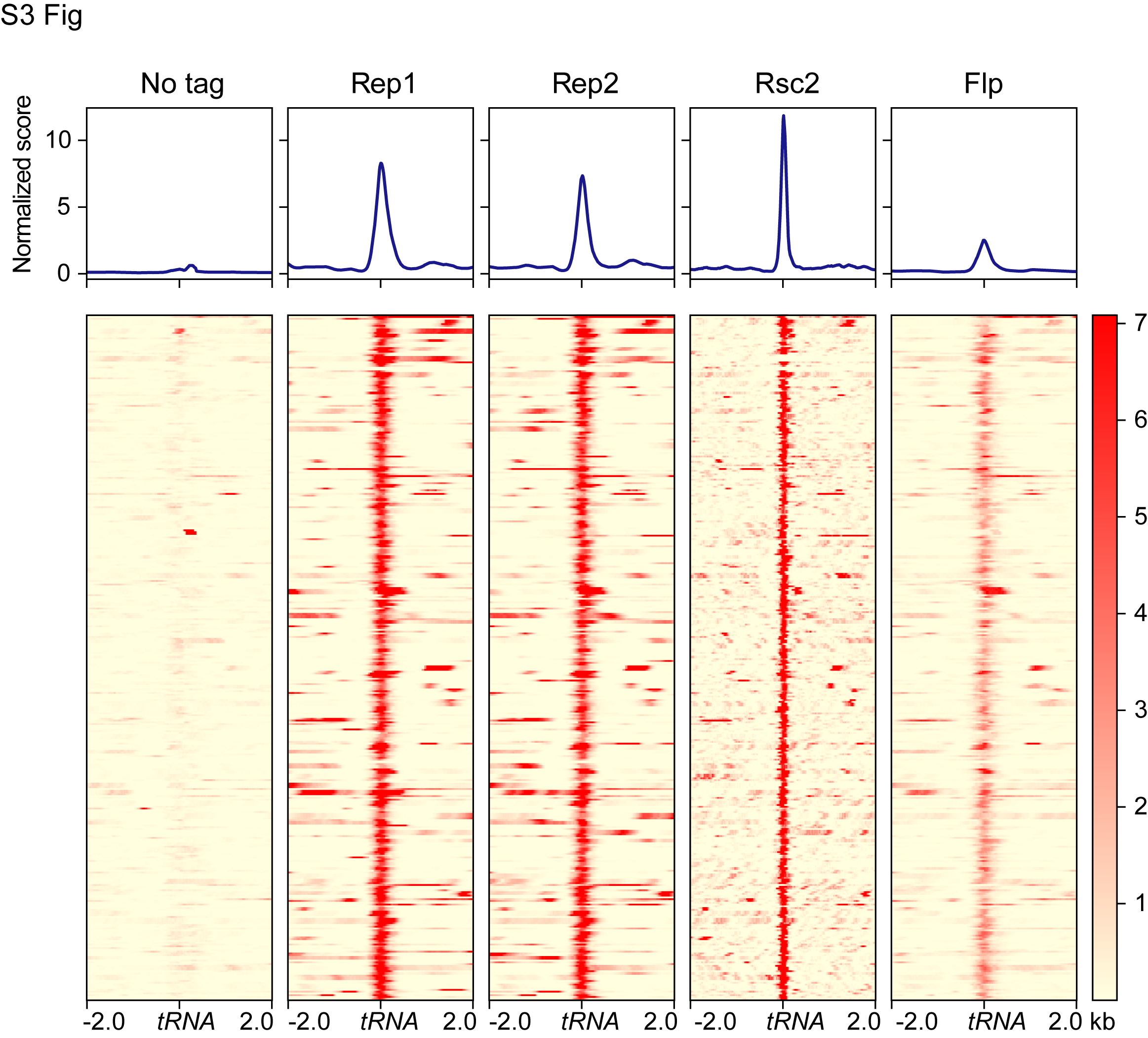

Supplement: S3 Fig — The top part of each panel shows the average binding profile and the bottom shows binding to each region as a heat map. (TIF) [file pgen.1010986.s005.tif]

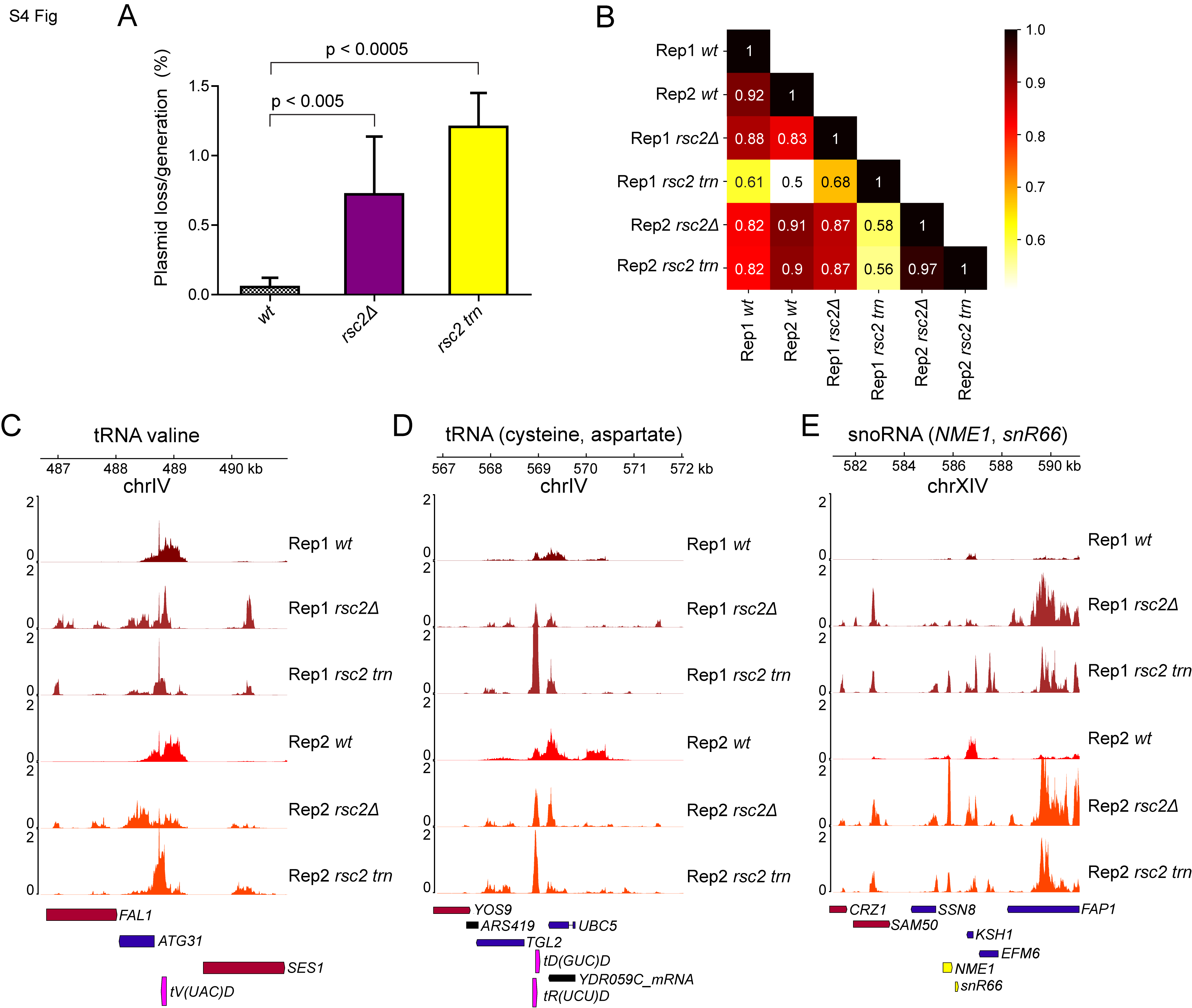

Supplement: S4 Fig — (A) Plasmid loss rate per generation, measured as described previously [83], is increased in both rsc2Δ and truncated rsc2 (rsc2 trn). (B) Genome-wide correlation of binding as measured by the ChIP-seq assay for Rep1 and Rep2 in the indicated strain backgrounds. Rep1 shows better correlation genome-wide with Rep2 in the wild type (wt) strain than with Rep1 in the rsc mutant strains, and Rep2 behaves likewise. (C-E) Rep1 and Rep2 localization at tRNA and snoRNA loci in wild type, rsc2Δ and truncated rsc2 strains as measured by ChIP-seq. In some instances, a redistribution of Rep1 and Rep2 can be seen around the original binding sites in the rsc2 mutant strains. (TIF) [file pgen.1010986.s006.tif]

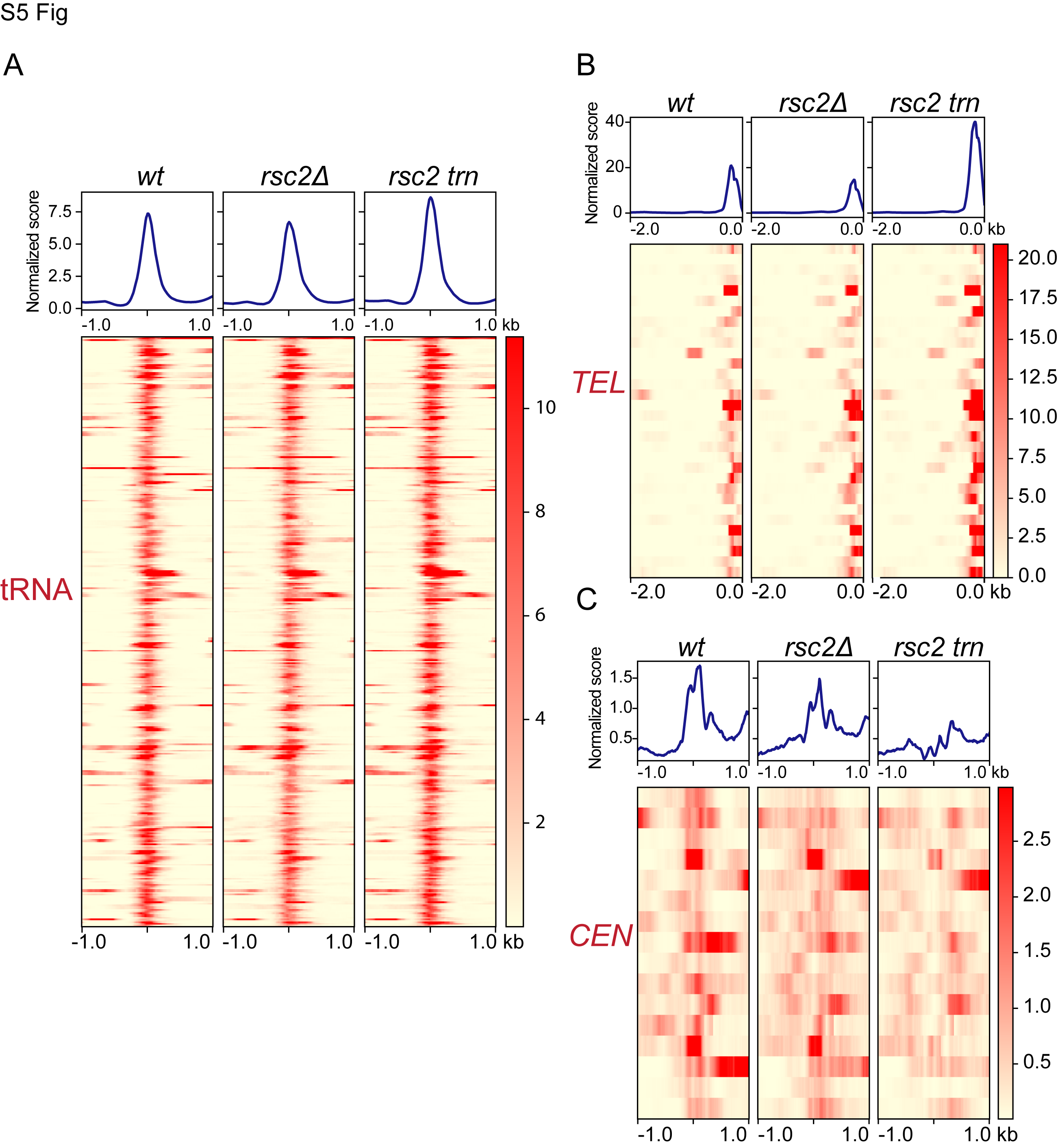

Supplement: S5 Fig — Binding of Rep1 at (A) tRNA loci, (B) TELs and (C) CENs as measured by ChIP-seq. (TIF) [file pgen.1010986.s007.tif]

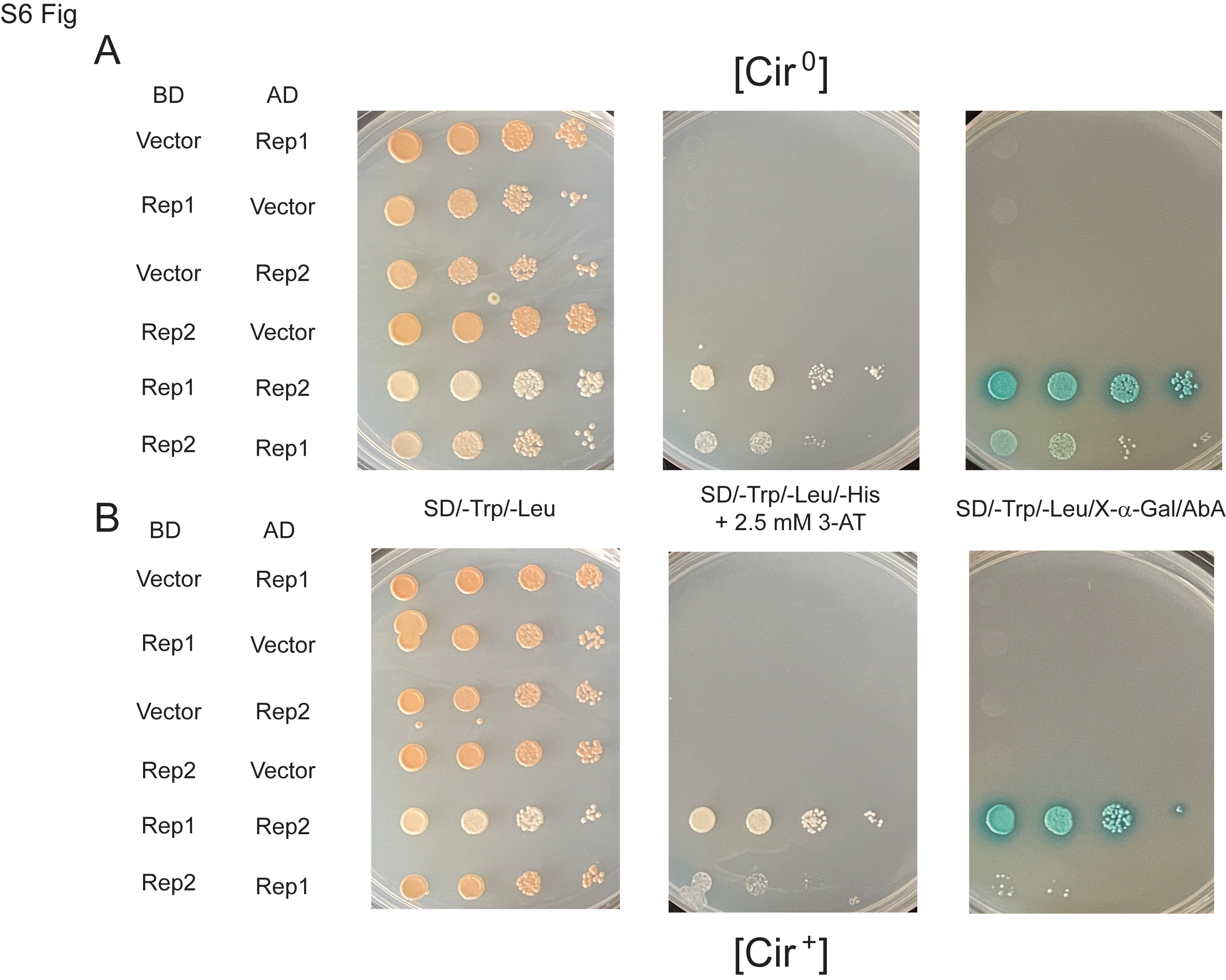

Supplement: S6 Fig — (A, B) The interaction between Rep1 and Rep2 tested here served as a positive control for the assays shown in Fig 7. This interaction was weaker with the [Rep2-BD]-[Rep1-AD] pair than with the [Rep1-BD]-[Rep2-AD] pair in both the [Cir0] and [Cir+] hosts. Presumably, the domain fusions in the former bait-prey configuration interferes partially with Rep1-Rep2 interaction. (TIF) [file pgen.1010986.s008.tif]

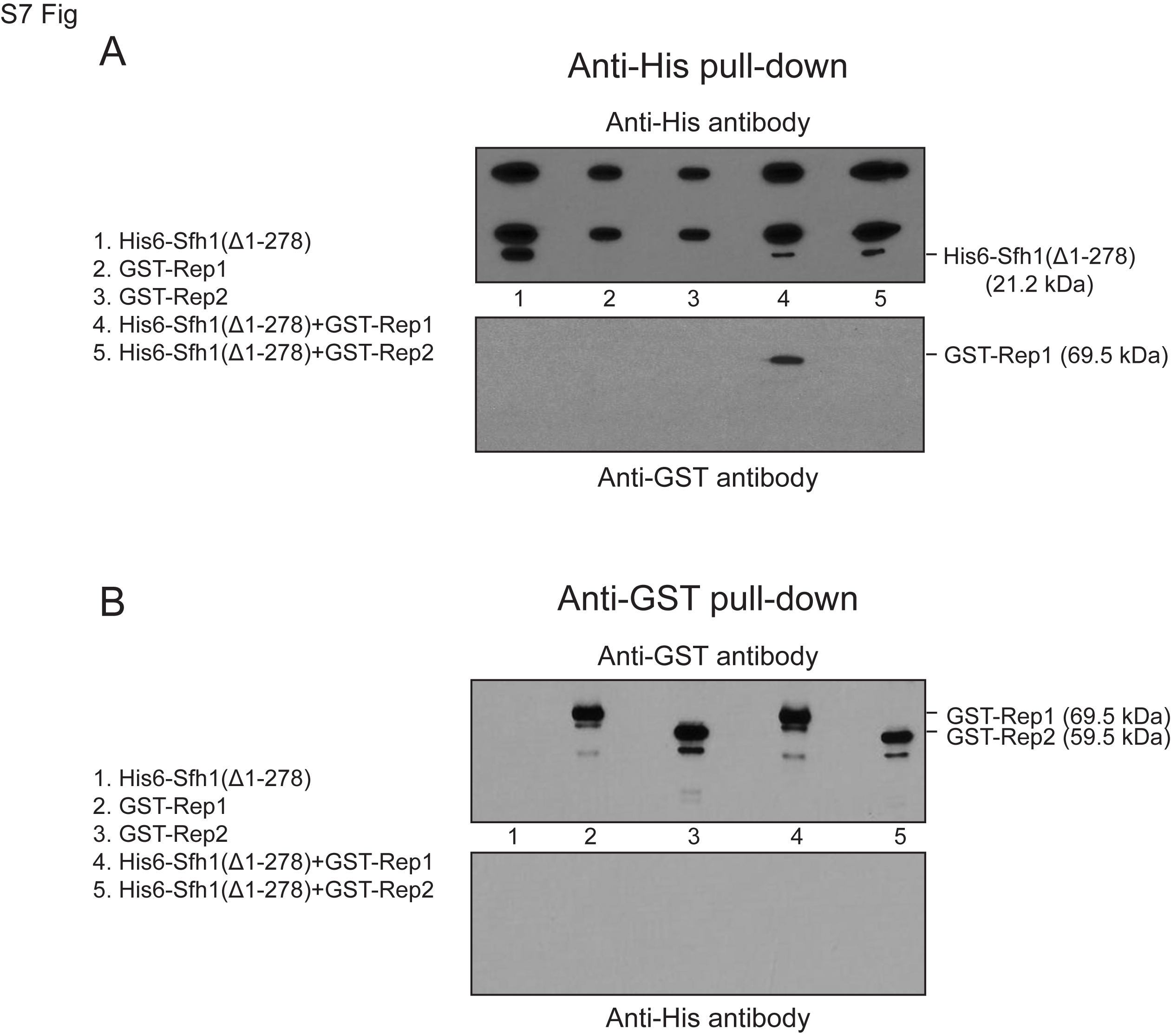

Supplement: S7 Fig — (A, B) The assays were similar to those shown in Fig 8, except that Sfh1(Δ1–278) was expressed instead of Sfh1. (TIF) [file pgen.1010986.s009.tif]

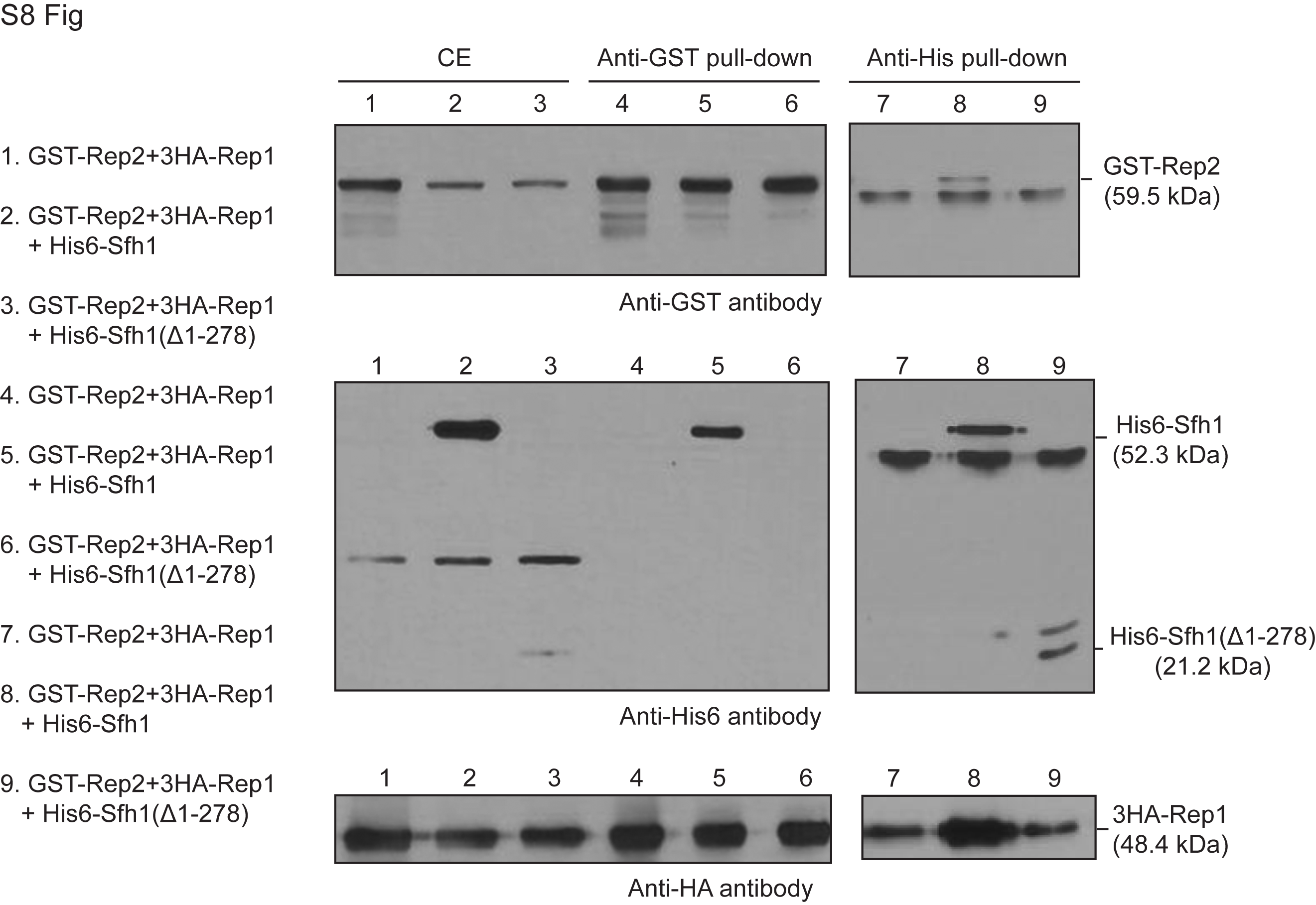

Supplement: S8 Fig — (A, B) The analyses were performed using extracts from E. coli strains expressing either Sfh1 or Sfh1(Δ1–278) together with both Rep1 and Rep2. The methodologies were analogous to those employed for the assays shown in Figs 8 and S7. (TIF) [file pgen.1010986.s010.tif]

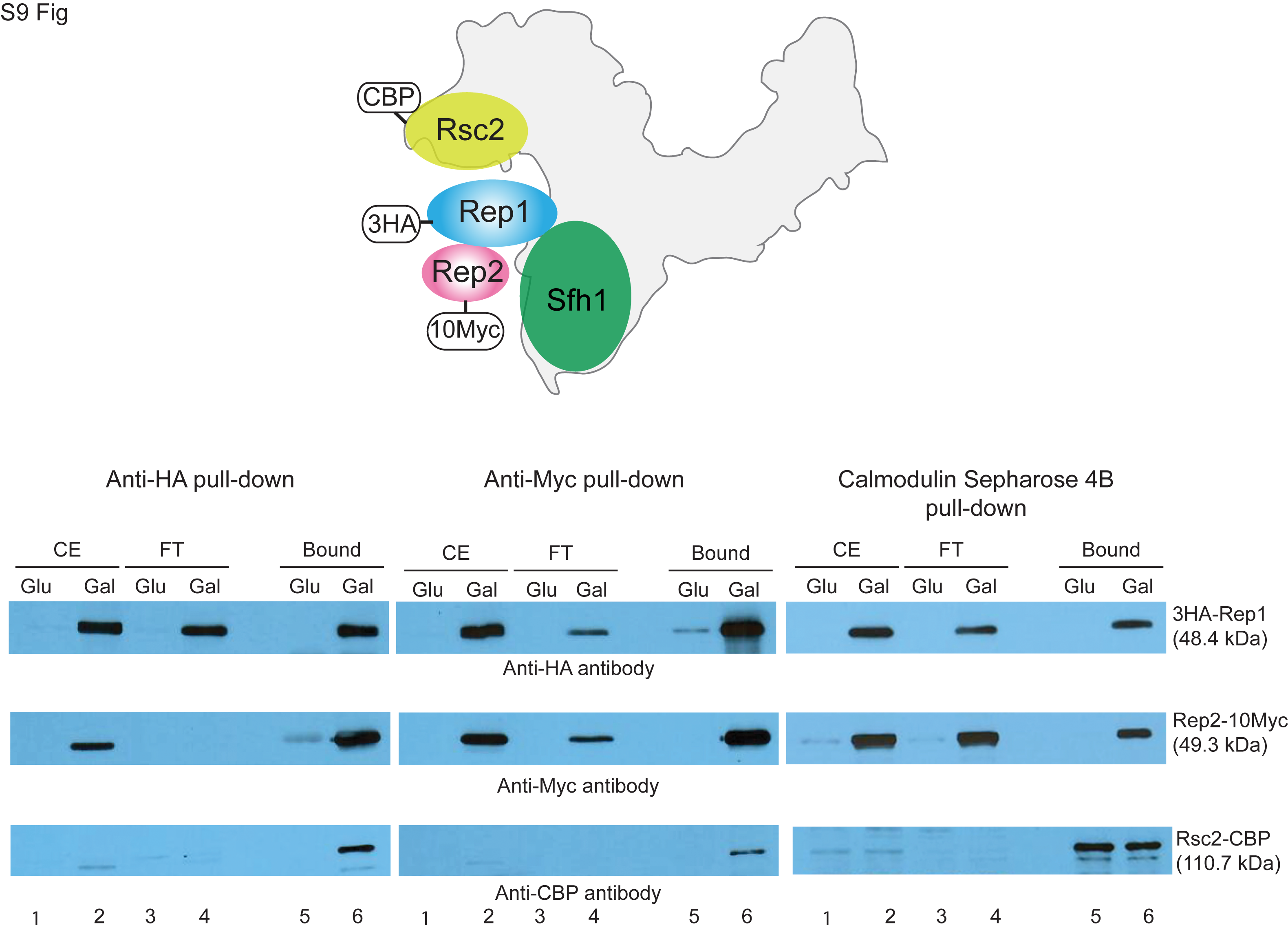

Supplement: S9 Fig — The schematic diagram depicting the potential interaction of the RSC2 complex with Rep1-Rep2 is modeled after the corresponding diagram in Fig 9. The Rsc2 protein derivative for this assay carried the CBP-tag without the accompanying dual Protein A-tag. The primary enrichment was performed using anti-HA (left panel), anti-Myc (middle panel) or anti-CBP (right panel) antibody. Western blotting was performed with each of these antibodies to test the co-enrichment of suspected partner proteins. (TIF) [file pgen.1010986.s011.tif]

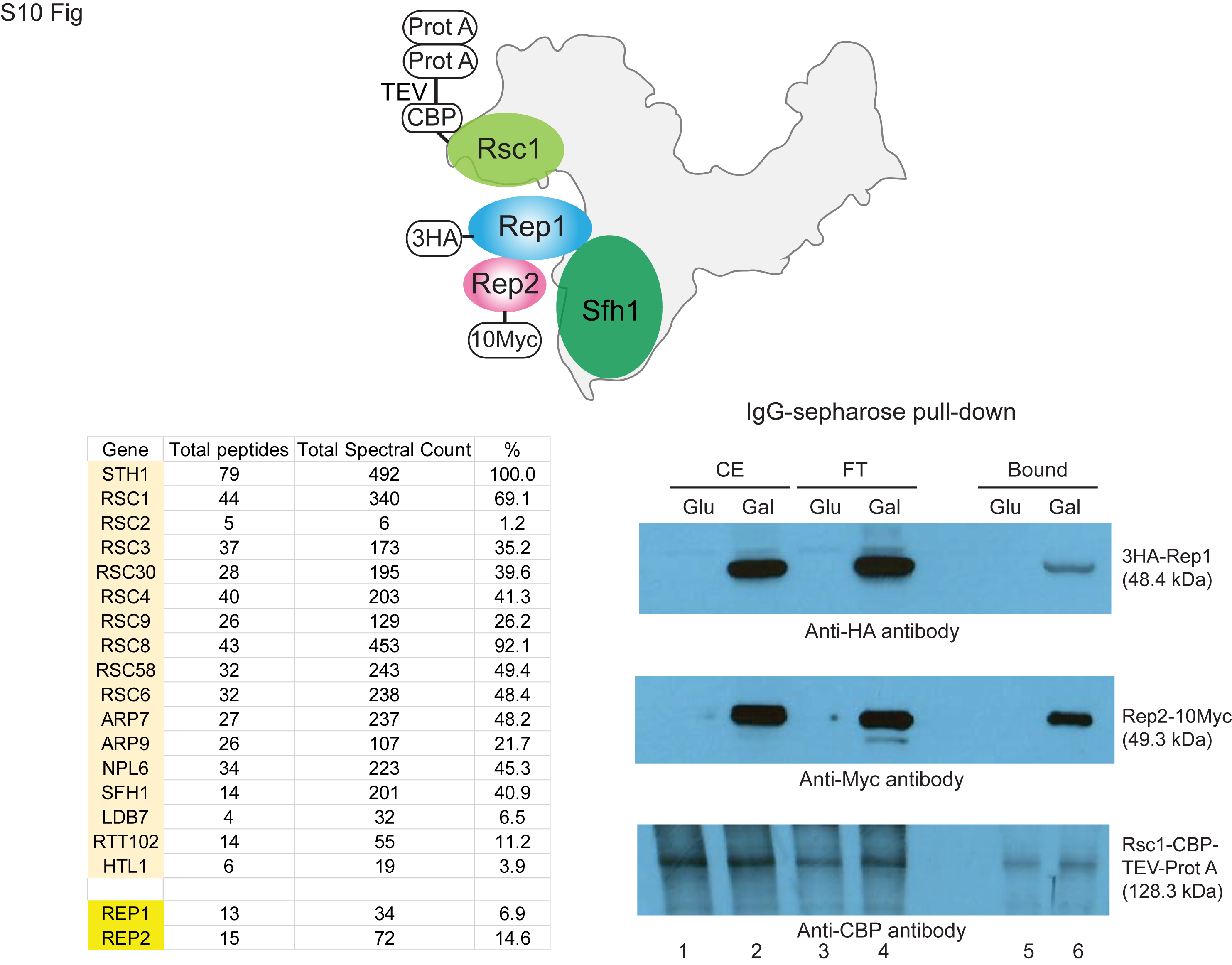

Supplement: S10 Fig — The schematic diagram (top) for Rep1-Rep2 interaction with the RSC1 complex is a close replica of that in Fig 9, with Rsc2 replaced by Rsc1. The epitope tags fused to Rep1, Rep2 and Rsc1 are indicated. The Table below at the left lists the relevant mass spectrometry data for the RSC1 complex obtained by enrichment on IgG-sepharose beads. The association of Rep1 or Rep2 with the enriched complex was probed using anti-HA or anti-Myc antibodies (directed to Rep1 or to Rep2, respectively) (right panel). (TIF) [file pgen.1010986.s012.tif]

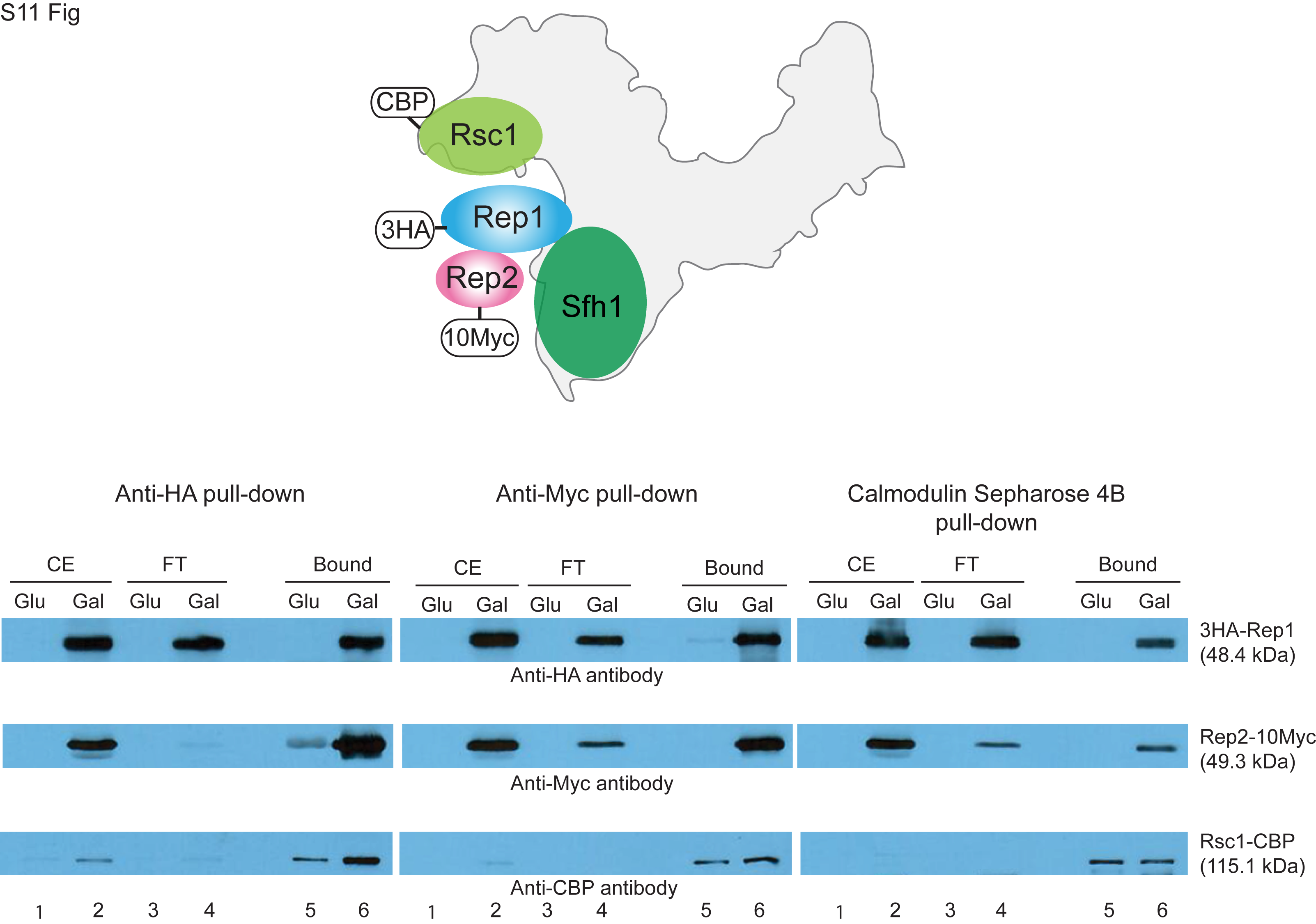

Supplement: S11 Fig — The schematic diagram from S9 Fig is redrawn here with Rsc1-CBP replacing Rsc2-CBP. Enrichment of individual proteins and probing for associated proteins by western blotting were carried out as in the assays depicted in S9 Fig. (TIF) [file pgen.1010986.s013.tif]

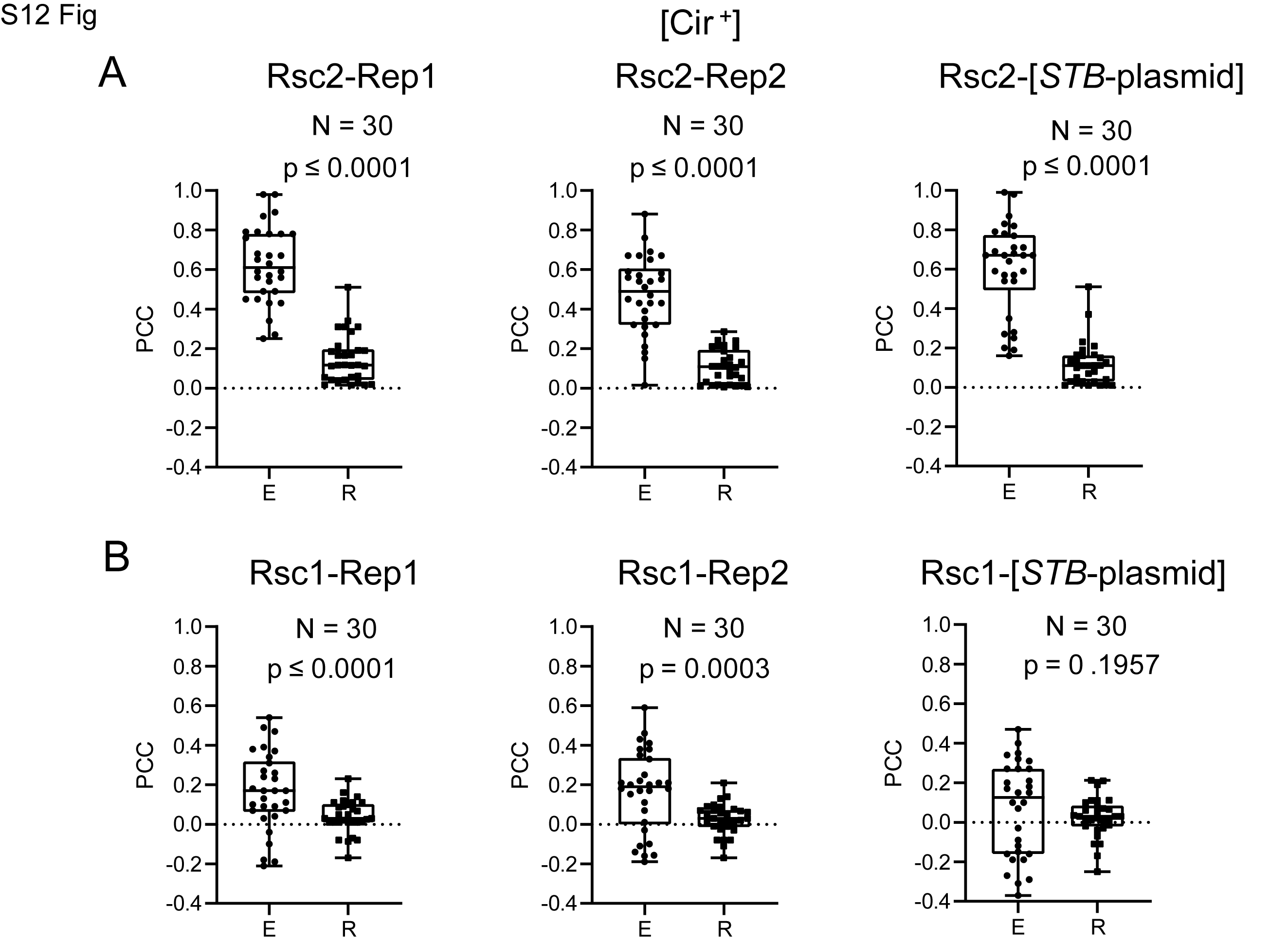

Supplement: S12 Fig — (A, B) The experimental protocols were as described under Fig 10. To obtain the degree of random overlap, PCC values were obtained for each spread after rotating the red and green fluorescence images through 90° relative to each other. Note that the median PCC values for experimentally observed (E) and randomized (R) fluorescence overlaps in all three plots in (B) are below 0.3, the lower threshold set for ‘partial overlap’ (see Fig 10). (TIF) [file pgen.1010986.s014.tif]

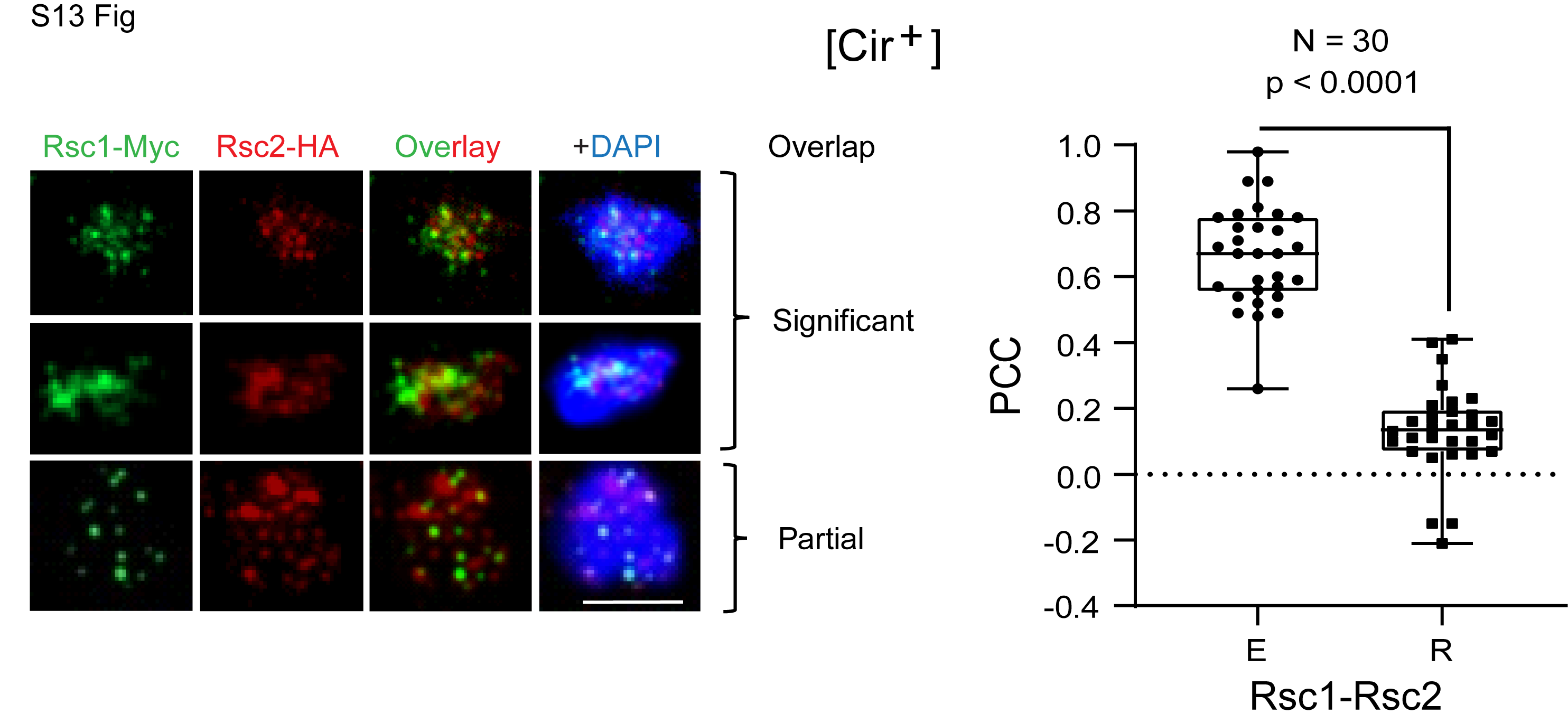

Supplement: S13 Fig — The epitope-tagged Rsc1 and Rsc2 were visualized by immunofluorescence in [Cir+] mitotic spreads. The extent of the experimentally observed Rsc1-Rsc2 fluorescence overlap (E) and the overlap following randomization (R) are plotted (see Fig 10). Bar = 5 μm. (TIF) [file pgen.1010986.s015.tif]

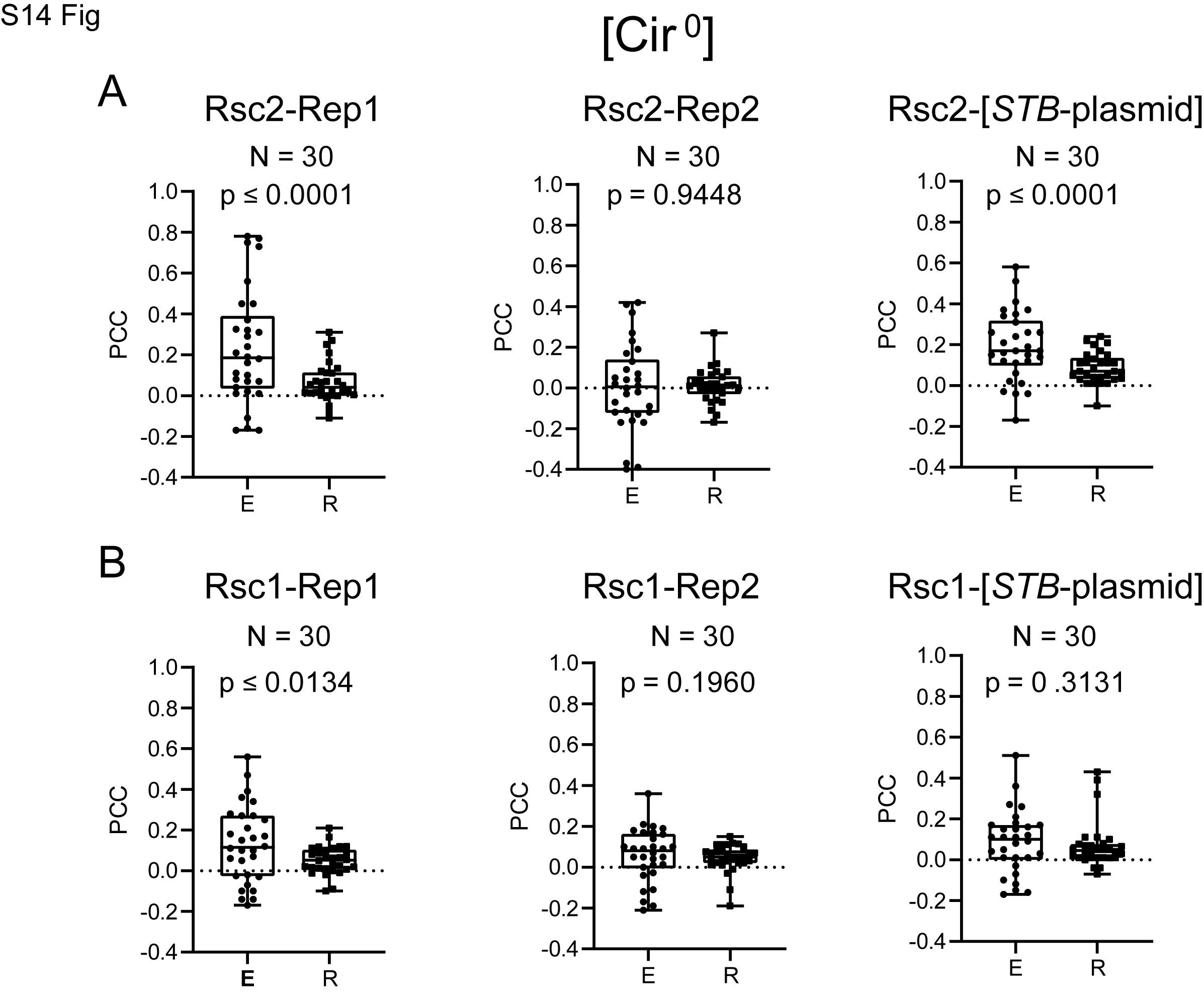

Supplement: S14 Fig — (A, B) The analysis was done as described in the legend to S12 Fig, except that [Cir0] chromosome spreads were assayed. The median PCC values for fluorescence overlap in the experimental (E) and randomized (R) samples in all the plots are < 0.3, signifying no overlap (see Fig 10). (TIF) [file pgen.1010986.s016.tif]

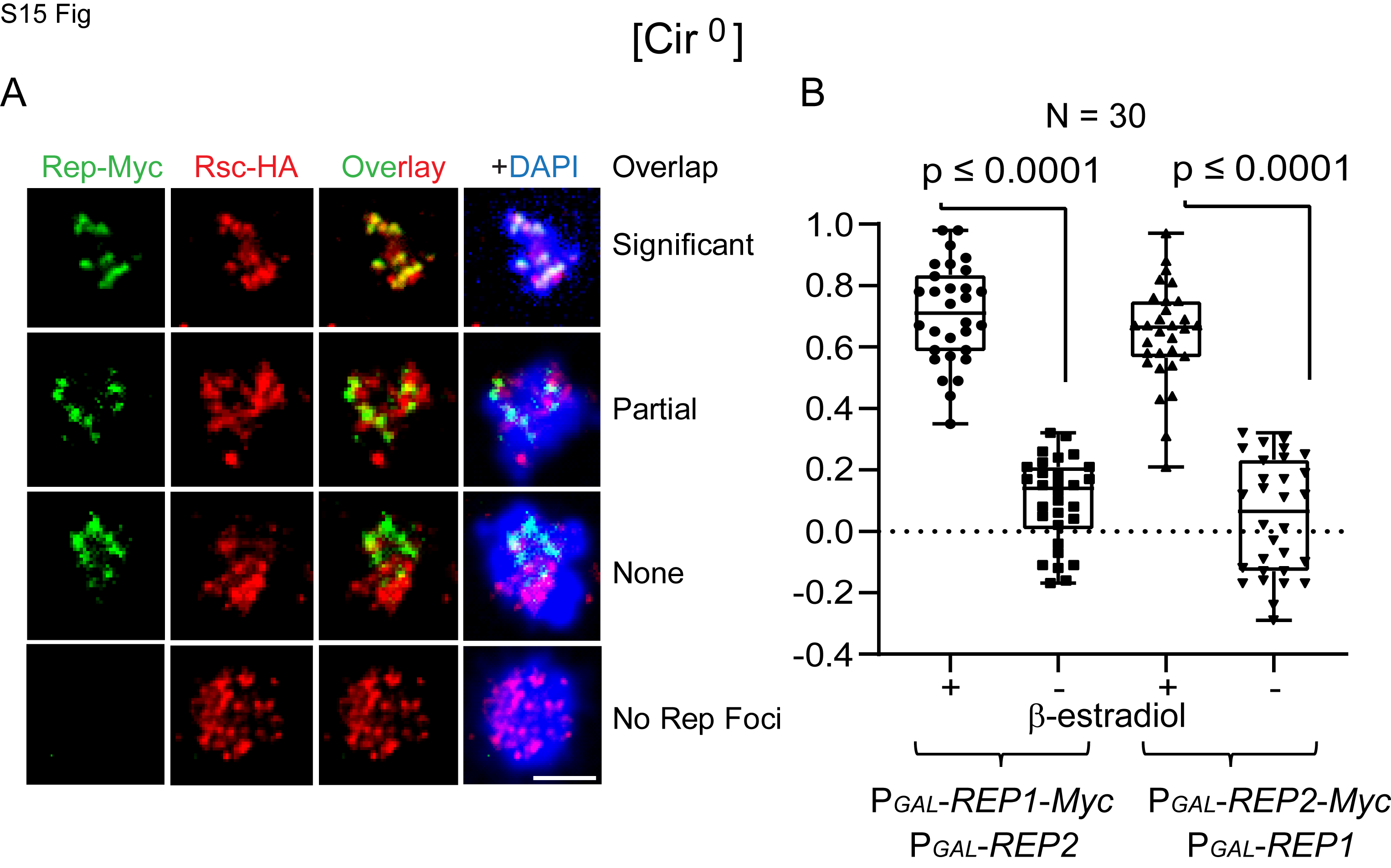

Supplement: S15 Fig — Chromosome spreads were prepared from [Cir0] diploid cells transferred to sporulation medium for 8 hr and were assayed by immunofluorescence microscopy. The native RSC2 locus was modified to express Rsc2-HA. Rep1-Myc or Rep2-Myc, expressed under GAL promoter control from an integrated cassette, was complemented by its untagged Rep partner expressed by a CEN-plasmid from the GAL promoter. A β-estradiol inducible activator system [98] was used to control the GAL promoter. Bar = 5 μm. (TIF) [file pgen.1010986.s017.tif]

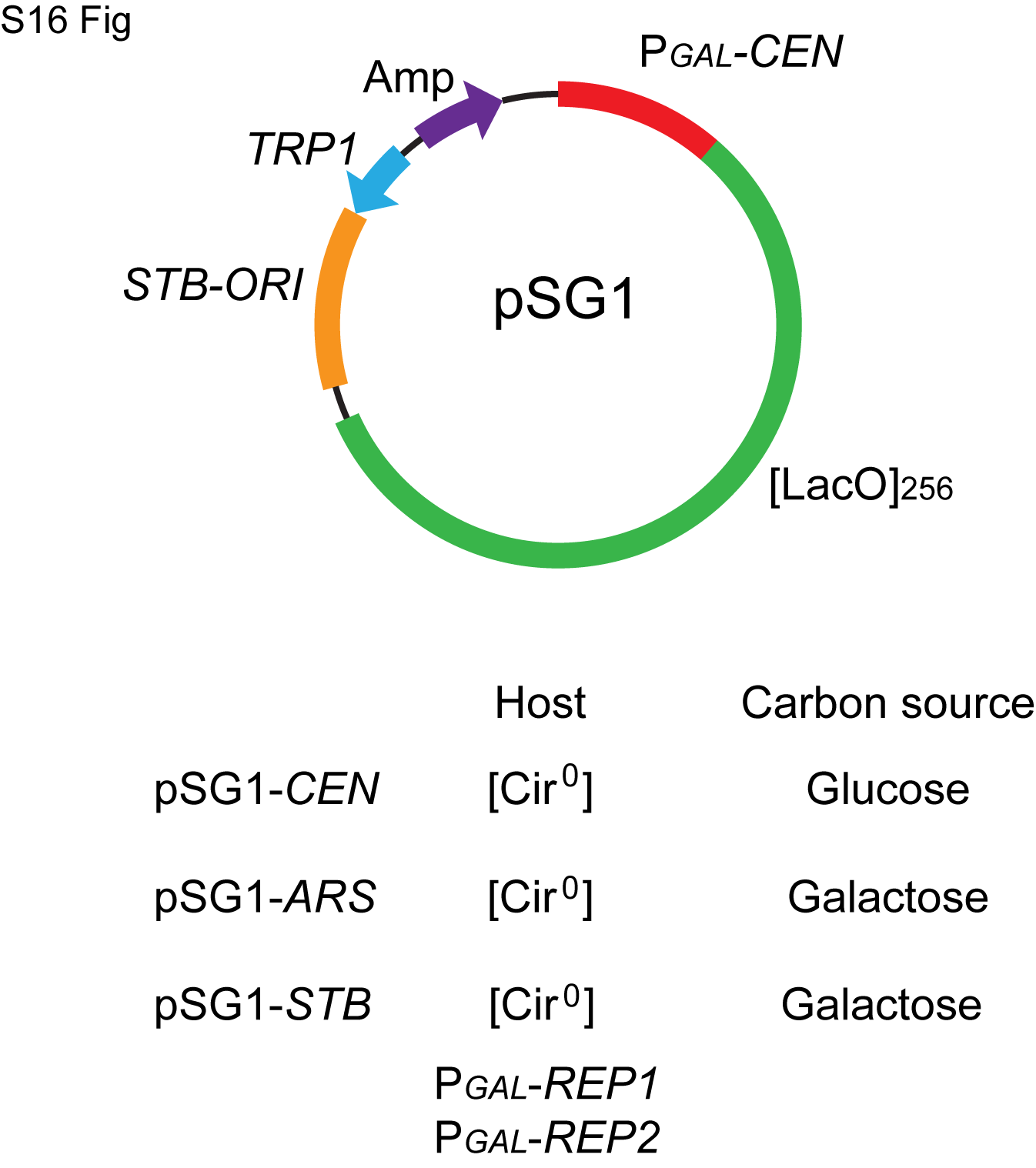

Supplement: S16 Fig — The features of the pSG1 plasmid are schematically diagrammed. In addition to the TRP1 marker for selection in yeast and [LacO]256, the plasmid carries the ORI-STB sequence from the 2-micron plasmid and a CEN sequence whose function is regulated by the GAL promoter [33]. The plasmid behaves as pSG1-CEN in a [Cir0] host grown in glucose (Rep1-Rep2 proteins absent; CEN active) and as pSG1-ARS when this strain is shifted to galactose (CEN inactive). In a [Cir0] host expressing Rep1 and Rep2 under GAL promoter control, the plasmid is pSG1-STB in the presence of galactose. (TIF) [file pgen.1010986.s018.tif]
