# Supplementary material for: The selfish yeast plasmid exploits a SWI/SNF-type chromatin remodeling complex for hitchhiking on chromosomes and ensuring high-fidelity propagation
Source: PLoS Genet. 2023 Oct 9;19(10):e1010986. doi: 10.1371/journal.pgen.1010986 (PMC10586699; doi:10.1371/journal.pgen.1010986)
Supplement: Attachment — Submitted filename: PLoS-Reviewer-Responses.docx [file pgen.1010986.s019.docx]

**Responses to Reviewer comments**

We appreciate the careful reading of the manuscript by the reviewers and their thoughtful comments. We have revised the manuscript to address all their comments, which we feel have improved the manuscript. In revising the manuscript, we have added one new supplementary figure with new data (S2 Fig) and moved two figures from the supplementary figures to the main manuscript. All figures have therefore been renumbered. Below we address each of their specific comments.

**Reviewer 1**

*1) A reasonable concern is that the association of Rep1/2 to RSC2 chromosomal binding sites (Figs 1-6, 8 as well as S9-S10) is an artifact of overexpression. The authors should acknowledge this caveat but then justify to the reader why the data is still admissible. Are there any experiments are is it merely the bulk of consistent data?*

We performed additional experiments to address this concern in two ways. First, we examined the steady state protein levels of Rep1 and Rep2 in strains where they were expressed from the *GAL*-promoter, in comparison to strains where they were expressed under the control of their native promoters from the 2 micron plasmid. Protein levels were comparable for Rep2 and slightly higher from the native promoter for Rep1, making it unlikely that expression from the *GAL* promoter causes widespread artifactual binding of Rep1 and Rep2 throughout the genome. We have now added this new western blot data for the two expression conditions (S2A Fig). Second, we examined the localization of Rep1 and Rep2 by ChIP-PCR at the preferred tRNA_val-UAC_ (tRNA valine) target locus, again comparing Gal induction with native promoter expression. The occupancy of the tRNA_val-UAC_ locus was also comparable (albeit qualitatively) between the two sets of strains, indicating that Gal overexpression alone is not responsible for the ChIP signal at this archetypal target locus. Furthermore, Rep1-Rep2 occupancy at a negative control locus (*TRP1*) was not above background under the two conditions. These new data have also now been included in the manuscript (S2B Fig).

Finally, we have added the following sentences to the penultimate paragraph of the first section of 'Results' (page 8-9 lines 151-159). "Because the epitope-tagged Rep proteins were expressed under the control of the *GAL1-10* promoter in these assays, we compared their expression under galactose induction with their native expression levels. The galactose-induced steady state level of Rep2 was comparable to that observed for its expression from the multi-copy 2-micron plasmid promoter (S2A Fig). For Rep1, the expression from the native promoter was stronger (S2A Fig) than, or similar to, that from the GAL promoter. Moreover, the occupancy of the *tV(UAC)D* (tRNA valine) locus (see Fig 1A) was comparable between the two sets of strains, indicating that galactose-induced overexpression alone was not responsible for the ChIP signal (S2B Fig)."

*2) The authors provide representative examples of Rep1/2 mapping in figure 1 (presumably the most convincing examples) and then follow with heat maps in subsequent figures. The data are initially described as providing “clear localization”/”consistently high signals” when the data are actually less than perfect. I believe it is a disservice to the reader to 1) not acknowledge the limitations up front and that 2) the data must be viewed in aggregate to draw conclusions. The authors eventually point to the substantial signal for the Flp control by CUT&Tag at CEN5 and TEL15 in the examples of figure 1. I believe it is more helpful to make this point with the heat maps where it is shown how different techniques support their conclusion better at different sites. Specifically in figure 2, the full magnitude of the Flp control signal by CUT&Tag can be appreciated but counterbalanced by the ChIP-Seq data. Additionally, figure 2 shows that the No Ab control for CUT&Tag for telomeres has the highest signal of all. This should be acknowledged.*

We used the phrase "clear localization" (page 7 line 128) in the context of Fig 1, which shows clear signal for Rep1 and Rep2 at the example sites in both the ChIP-seq and CUT&Tag assays. We used the phrase "consistently high signals" later on (page 9 line 160), subsequent to our description of the variable background signal with the Flp control at *CEN*s and *TEL*s in the CUT&Tag assay. We do not believe that readers will be misled by our presentation of the data  and description of the results. The reviewer's point about the No Ab control at *TEL*s is well taken. However, we believe that the apparent strong average signal in the No Ab control at *TEL*s (Fig. 2A) reflects a small number of *TEL*s. At the majority of *TEL*s where there is notable Rep1 signal, the signal from both the No Ab control and Flp control is lower as seen in the heatmap (Fig. 2A). We now explicitly acknowledge this in the revised manuscript by adding the sentence (page 8 lines 142-146) "At *TEL*s, the no antibody control in the CUT&Tag assay showed apparently higher average signal than Rep1; however, this is caused by strong background signal at a small number of *TEL*s. At most *TEL*s where there was any appreciable Rep1 signal, it was higher than the controls (Fig 2A)".

*3) While the bulk of tRNAs may localize to the CEN cluster or nucleolus, some tRNAs do not localize to either (Belagal Gadal 16; Chen Gartenberg 14). The localization of the tRNA in the colocalization study of figure 9 must be known to draw the conclusions the authors state. When the plasmid is near the tRNA, is the tRNA near CENs or the nucleolus. This would be reasonable to test or the interpretation should be modified. Additionally, some of the measurements are made in glucose while others are made in galactose, which induces changes in genome organization (Brickner lab and others) that could affect the results. In addition, why choose this tRNA gene? Did it have a strong mapping signal?*

We analyzed published Hi-C data (Duan et al Nature 2010) to examine the associations of the tRNA valine gene (tRNA_val-UAC_ at chrIV: 488797) that was used in Figure 9 (now Figure 11) as well as in Figure 1A and S2 Fig. The Hi-C data indicate that this tRNA valine locus is not associated with the nucleolus but is associated with several *CEN* loci, indicating that it is part of the *CEN* cluster. This association is entirely consistent with all our data including Figure 11, showing that pSG1-*CEN* foci are in closer proximity to the tRNA valine gene than pSG-*STB* foci. These data and analysis are also consistent with the physical proximity of this tRNA locus to *CEN IV* (~39 kb), as Pol III transcribed genes located near *CEN*s are apparently blocked from associating with the nucleolus (Belagal P et al, Mol. Biol. Cell. 2016)

We have revised the relevant section of the Figure 11 results for clarity, which now reads as follows (page 18, lines 362-374).

"Analysis of published Hi-C chromosome conformation capture data reveal that the tRNA_val-UAC_ locus, which is about 39 kb from *CEN IV*, is part of the *CEN*-proximal tRNA gene cluster and not the nucleolar tRNA cluster [57], consistent with a block in pol III-transcribed genes near *CEN*s from associating with the nucleolus [58]. Tighter association of pSG1-*CEN* than pSG1-*STB* with the tRNA_val-UAC_ locus is expected, as tRNA loci are only one set of preferred tethering sites for pSG1-*STB*. By contrast, any association of pSG1-*ARS*, which does not localize to chromosomes, with tRNA genes can only be a chance occurrence.

The pSG1-*CEN* plasmid foci were in closer proximity to the tRNA_val-UAC_ locus than pSG1-*STB* foci, as indicated by the median distance plots (Fig 11). The cluster of tRNA loci containing the tRNA_val-UAC_ gene would be associated with the single *CEN*-cluster, which includes the pSG1-borne *CEN* as well."

Although pSG1-*CEN* and pSG1-*STB* were evaluated in glucose- and galactose-grown cells respectively, given that pSG1 is an extrachromosomal plasmid, there is no reason to think that any changes in overall genome organization would affect the association of pSG1 with the tRNA valine locus. Based on our previously published results (Ma et al., NAR 2013; Liu et al., NAR 2016), a single-copy *STB*-reporter plasmid can be stabilized to similar extents (~70%) by providing Rep1 and Rep2 from the endogenous 2-micron plasmid (40-60 copies per cell) or by expressing these proteins under ADH promoter or *GAL* promoter control from single copy expression cassettes (see bars outlined in green in the figure below). Thus, potential modulations in chromosome organization due to changes in carbon source do not appear to have a perceptible effect on plasmid physiology.

Ma et al. NAR 2013, 41:2340-2353, Fig.6 Liu et al. NAR 2016, 41:8302-8323, Fig. 5

The tRNA_val-UAC_ (tRNA valine) locus was chosen because it showed good localization of Rep1 and Rep2 by ChIP-seq (Fig 1A; labeled *tV(UAC)D*).

*4) Figure 10 provides a proof-of-principle that tethering a plasmid to RSC2 confers segregation. My concern is that similar results might be obtained by tethering the plasmid to any chromosome bound protein. If so, then this experiment tells us that hitchhiking can occur but does not inform on whether RSC normally carries out this function. Are there classes of proteins for which this would work and those for which it would not?*

The principal conclusion from the collective evidence presented in this manuscript is that Rep1-Rep2 serves as a protein bridge between an *STB*-plasmid and the RSC2 complex. We do not rule out the possibility that tethering the plasmid to some other chromosome-bound protein could improve its stability. Such a result, taken in isolation, would not constitute proof that such a protein is normally responsible for hitchhiking. It is important to note that our study combines several lines of evidence, including 1) the overlap of RSC2 and Rep protein localization sites on chromosomes, 2) the requirement of RSC2 for plasmid stability, 3) the molecular interaction of Rep proteins and a member of the RSC2 complex, 4) the overlap of Rep proteins, plasmid and RSC2 in yeast chromosome spreads using fluorescence assays, followed finally by 5) improving chromosome association and stability in the absence of Rep proteins by artificially tethering the plasmid to RSC2. The purpose of this final experiment (now Figure 12) was to test whether Rep1-Rep2 could be bypassed by an alternative protein bridge between a plasmid and the RSC2 complex to reconstitute segregation by chromosome hitchhiking. We emphasize that the rationale behind Figure 12 follows the establishment that the RSC2 complex is an authentic mediator of 2-micron plasmid-chromosome association by the criteria listed above. The possible outcome that tethering a plasmid to another chromatin protein improves its stability does not undermine our findings in any way.

Whether there are classes of chromatin proteins for which the tethering experiment would give a positive result and those for which it won't is hard to tell, but presumably, yes. The efficiency of segregation may vary depending on individual chromatin binding proteins.

To avoid possible misimpression of our reasoning, we have now revised the title of the section describing Fig. 12 under 'Results'. The new title is 'A Rep1-Rep2 bypass system for plasmid association with the RSC2 complex promotes segregation by chromosome-hitchhiking'.

Minor comments

*1) While pericentric chromatin may be unusual in yeast, I am not aware of it satisfying heterochromatic criteria. Do the Sir proteins silence genes there?*

The reviewer is correct that the silenced chromatin at *CEN*s in *S. cerevisiae* does not meet the standard criteria for heterochromatin, unlike *TEL*s. Whereas Sir1 is present at both centromeric and silent chromatin in budding yeast, Sir2-4 are associated with only silent chromatin (Sharp et al, 2003, Genes & Dev 17: 2356-2361). However, the nucleosome assembly factors CAF-1 and Hir proteins contribute to both silent and centromeric chromatin (Sharp et al., 2002, Genes & Dev 16: 85-100).]. We have revised the sentence describing the propensity of Rep proteins to associate with these loci (page 7 lines 121-123) as follows.

"It is notable that *TEL*s and *CEN*s are typically silenced or transcriptionally quiescent chromatin whereas tRNA and snoRNA loci are highly transcribed chromatin."

*2) Figures S1 and S2 were less ambiguous to me than the current Figure 1. The authors must have some reason for delegating them to the supplement.*

Figures S1 and S2 (now Figure S3 in the revised manuscript) show an overview of Rep protein localization at large chromosomal regions (S1 Fig) and at tRNA loci (S3 Fig), together with controls, using only the ChIP-seq assay. Figure 1 shows examples of binding to many distinct types of chromosomal loci, using both the ChIP-seq and CUT&Tag assays and also include control samples. We did not think there was greater ambiguity with either set of figures, but since Figure 1 is a broader representation of the overall data, we feel that it's appropriate in its place.

*3) In summarizing the initial mapping data, the authors suggest that plasmids might not be tethered to all sites because there are only a small number of plasmid foci in dividing cells. I am not certain I understand the argument. CENs, TELs and tRNAs all cluster so might the plasmids be localized to these clusters, thus yielding occupancy at all sites? Or is it that only a subset of the hundreds of sites are occupied in a single cell but that the population average makes it appear as if the 100s of sites are always occupied?*

Although Rep1 and Rep2 have a tendency for preferential localization at *CEN*s, *TEL*s and tRNAs (clustered loci), there are also hundreds of other (less preferred) chromosomal Rep1-Rep2 binding sites that do not form clusters. Therefore, the 3-6 plasmid foci in a given cell would be present only at a subset of all possible Rep1-Rep2 occupancy sites mapped at the population level by ChIP-seq or CUT&Tag. These bulk assays cannot distinguish whether all possible Rep1-Rep2 occupancy sites are filled by these proteins in every cell or only subsets of the cumulative set of sites mapped in the population are occupied in individual cells.

*3) Does the Rsc2-truncation mutant retain some remodeling activity? If not, does the residual signal of Rep1 binding in this mutant (Fig 4) indicate that recruitment is independent of remodeling? I think that would be a significant claim.*

It is not known whether the Rsc2 truncation retains remodeling activity, as that would depend on the integrity and function of an RSC2 complex containing the truncated Rsc2 protein. Presumably the RSC1 form of the complex will retain remodeling activity in this mutant strain. We also do not know how the recruitment of Rep1 via the RSC2 complex depends on its remodeling activity, as our experiments do not address the link between remodeling and recruitment.

*4) Either there is a typo in the y-axis label of figure 10A (the mis-segregation panel) or I do not understand the description in the figure legend.*

In this figure (now Fig 12A), there are two plots showing the effect of tethering the plasmid directly to RSC2 via Sfh1-LacI. In the plot on the left, the Y axis indicates the percentage of cells with equal (1:1) segregation between mother and daughter cells, and it shows that tethering via Sfh1-LacI significantly improves equal segregation. In the plot on the right, the Y axis indicates the frequency of missegregation into the daughter cell (0:2), as a fraction of *all* missegregation events (the sum of missegregation into the daughter and missegregation into mother). The proportion is (0:2)/[(0:2) + (2:0)], which is indicated on the Y axis. This plot shows that mother bias, which is the default condition in the absence of a partitioning system, is significantly reduced by tethering via [Sfh1-LacI]-[LacO-plasmid].

**Reviewer 2**

**General**

----- *“Although the authors provide new data, the main observations presented in the manuscript are already published. [Rep1-Rep2]-RSC interaction is shown in Ma et al., Nucleic Acids Research, 2013 (reference 35 in the manuscript) and the requirement of RSC2 for Rep1 localization is shown in Wong et al., Mol Cell Biol, 2002 (reference 36 in the manuscript).”*

This characterization by the reviewer regarding the novelty of the manuscript would seem to be rather uncharitable. The Wong et al. and Ma et al. papers referred to here, while showing the requirement of the RSC2 complex for 2-micron plasmid segregation, did not address the mechanism of its action. The altered nucleosome organization at *STB* in the *rsc2Δ* strain reported by Wong et al. would be consistent with a number of possibilities—(**a**) regulation of Rep1 expression from its promoter and (**b**) modulation of partitioning factors recruited at *STB*, among others. The Ma et al. work demonstrated the ability of affinity-tagged Rep1/Rep2 to pull down components of the RSC complex. Given the multiple interaction partners of Rsc2 and other proteins of the RSC2 complex, the functional significance of Rep-RSC interaction in plasmid partitioning was unclear. In light of the Wong et al. finding, one possible interpretation was that Rep1-Rep2-dependent recruitment of RSC2 at *STB* and RSC2-promoted remodeling of *STB*-chromatin were required for stable plasmid segregation. Our present study has now taken Wong et al. and Ma et al. to the next level by exhaustively characterizing Rep-RSC2 interaction and establishing its role in colocalizing Rep1-Rep2 (and thus an associated *STB*-plasmid) at chromosomal sites bound by RSC2. The altered nuclear localization of Rep1 under *rsc2Δ* suggested by Wong et al. is not comparable in resolution to the genome-wide mapping of Rep-Rep2 accomplished in the present studies. In sum, mapping of chromosome sites utilized for chromosome-hitchhiking by the 2-micron plasmid and characterizing the interactions between the plasmid partitioning proteins and the RSC2 complex responsible for plasmid-chromosome bridging, in our opinion, represent a marked advancement from the current state of knowledge on the stable propagation of this selfish genetic element. Finally, we note that Reviewer 1 does not share the same view as Reviewer 2 as conveyed in *“----I believe this work adds significant mechanistic understanding of how an extrachromosomal selfish genetic element persists in yeast.”*

*- The figures are not well organized. For example, the authors use three main figures to depicture the genomic location of Rap1 and Rap2 and three additional figures to show how it changes in rsc2Δ and Rsc2 truncation. These six figures could be condensed in one. Also, all the pull-down experiments are in supplementary figures while just a two-hybrid assay is using a main figure.*

The current organization of the figures resulted from our desire to limit the number of main figures to 10. Furthermore, the journal format permits ready access to supplementary figures associated with a main figure. It is not clear to us how, as suggested by the reviewer, six data-intensive genomics figures (each with multiple panels), could be compressed into one without seriously compromising its readability. In response to the comment regarding pull-down experiments we have now moved two of the relevant figures to main set: S6 Fig showing Rep1-Sfh1 interactions by co-IP (now Fig 8) and S9 Fig showing RSC2 purification followed by mass-spectrometry/western blotting to reveal in vivo interactions with Rep1 and Rep2 (now Fig 9).

- *Rep1 and Rep2 genomic location obtained by from ChIP seq and CUT&Tag show discrepancies and, in some cases, the signal is not above background (especially in the case of Rap2). It can be informative to subtract the background and examine which peaks overlap in the two techniques.*

We are not sure which discrepancies in Rep1 and Rep2 genomic localization the reviewer is referring to. We did note some discrepancies with regard to how the Flp negative control behaved in the ChIP-seq and the CUT&Tag assays in our description of those results (see p 8 and response to Reviewer 1 above). It is important to note that although the two assays are independent, ChIP-seq is considered to be a standard assay for identifying genome-wide DNA protein interactions in vivo. We primarily used CUT&Tag as a secondary assay to bolster confidence in the ChIP-seq results at active promoters like tRNAs where there is a risk of seeing artifactually high signal from ChIP. As we noted in the Results, at active tRNA and snoRNA loci, the Flp control tended to show low signal in both the ChIP-seq and CUT&Tag assays compared to the Rep1 and Rep2 signals. Because of the inherent variability of the control ChIP or CUT&Tag signals at different classes of sites, we found that using the control Flp sample as the background and subtracting it causes a large variability in the final signal. Moreover, we do not actually utilize the peaks called by MACS2 in any of our analysis, which is instead presented as continuous average profiles and heatmaps over sets of genomic loci.

*- The differences in Rap1 and Rap2 localization in rsc2Δ and Rsc2 truncation should be confirmed by a quantitative technique such as ChIP-qPCR.*

This point is well taken. As all of the Rep1-Rep2 occupancy sites are not affected the same way (qualitatively and quantitatively) by *rsc2* deletion/truncation, the suggested ChIP-qPCR assay could be misleading for a subset of these sites. In particular, the effect of rsc2 truncation is to delocalize Rep1-Rep2 around their normal binding sites at several chromosome locations rather than eliminate or markedly decrease binding. An alternative to ChIP-qPCR, for quantifying the impact of *rsc2Δ* and *rsc2* truncation on the localization of Rep1 and Rep 2, is to measure the genome-wide correlations among the ChIP-seq datasets. We show in the plot below the low correlation between the wild type and mutant strains in chromosome localization of Rep1-Rep2, revealing the adverse effects of the rsc2 mutations. Specifically, Rep1 shows better correlation genome-wide with Rep2 in the wild-type strain than with Rep1 in the *rsc* mutant strains, and Rep2 behaves likewise.

*- Mass spectrometry data should be made available in an open access repository.*
These data have now been placed in an open access repository and the accession number has been included in the Data Availability section of the manuscript.

*- I consider than the most interesting part of the manuscript is the artificially binding of an ARS plasmid to Sfh1 to achieve equal segregation. The authors could use this system to search for additional Rep1-Rep2 interaction partners with relevance in plasmid segregation.*

This is an interesting suggestion. The assay will have to be retooled into a high-throughput screen to accomplish this goal. However, as we note in our response to Reviewer 1, comment #4, identifying another yeast protein that improves the stability of an ARS plasmid by artificial tethering, in and of itself, would not be evidence for a new factor involved in plasmid stability in the absence of all the other lines of evidence we provide in this study. Moreover, such a protein would not necessarily be a bona fide Rep1-Rep2 interaction partner.
